# Reconsidering intrapersonal communication through an interdisciplinary lens

**DOI:** 10.3389/fpsyg.2025.1569493

**Published:** 2025-08-11

**Authors:** Constance Bainbridge, Gregory Bryant, Rick Dale

**Affiliations:** Department of Communication, University of California, Los Angeles, Los Angeles, CA, United States

**Keywords:** intrapersonal communication, consciousness, mindfulness, wellbeing, self-talk

## Abstract

Intrapersonal communication is a classification of phenomena such as self-talk and imagined interactions in which communicative messages are contained within a single individual and internal systems comprise both sender and receiver roles. Historically, the construct has met criticism with objections rooted in the notion that intrapersonal communication is simply a form of social cognition, but not all self-communicative behaviors have clear or empirically defined social cognitive connections. Self-directed behaviors, from inner speech to mindfulness, permit individuals to shape and interpret their experiences. Empirical examination of these phenomena would benefit from a unified framework. Relevant work is discussed supporting the perspective that intrapersonal communication constitutes a valuable interdisciplinary classification, including early research, functional and developmental approaches, and current connected methodologies and their limitations. A theoretical model is proposed that can guide understanding of the boundaries of intrapersonal communication by characterizing sender and receiver roles in the intrapersonal interaction based on active and inactive status.

## 1 Introduction

As you begin your day, you may first think through a to-do list of action items. Perhaps you have a meeting and find yourself imagining an interaction you expect to have there, rehearsing possibilities and considering how your words may be interpreted. Or you may simply imagine a sequence of viable conversational topics. Whether this sequence has an imaginary listener or not may be irrelevant—you are merely brainstorming to yourself. You may have many ideas that you can sense, but they fail to take form until you express them internally with language. These mindful moments, and perhaps other behaviors and experiences, have at times been referred to as *intrapersonal communication*. In intrapersonal communication, we engage in information exchange within ourselves in ways that may influence our own actions or thoughts. This concept applies communication theories to processes that unfold within a single individual. A simple and provisional definition is that it involves information generation and transmission such that sender and receiver are the same cognitive agent.

The study of *inter*personal communication has long been influenced by the sender-receiver, or code model (Shannon, 1948). This familiar formulation frames communication as a sender transmitting a message to a receiver through a noisy channel. The sender has the intention of influencing a receiver of this message to provoke some understanding or action, the receiver decodes the message, and from there communication continues (e.g., Schramm, 1954). Later formulations included various features that might impact communicative action, including social context, emotions of the participants, and different modalities working together. There have long been challenges to this paradigm across many disciplines influenced by the sender-receiver model, including basic questions such as whether animal signals have inherent meaning, or to what extent language explicitly encodes speaker meaning (e.g., Gernert, 2006; Rendall et al., 2009; Sperber and Wilson, 1996). Despite these challenges, Shannon's multiparty format, with separate senders and receivers, comprises the defining criteria of the term “communication” across a wide variety of disciplines.

Intrapersonal communication seems like an *edge case* for the general concept of communication, because it challenges Shannon's multiparty distinction. Debates about how to define communication generally can be framed using edge cases that go beyond conventional domains of study (e.g., Cushman and Whiting, 1972; Craig, 1999). An example edge case is whether we should include communication with the self as consistent with a definition of communication. Outside of social interactions, we often experience a process akin to communication: we engage in verbal thought, speak aloud in isolation, write in journals, and so on. While many of us can relate to self-directed communicative behavior, the literature on the topic remains sparse and largely disjointed. Indeed, there is no unifying standard to encapsulate these behaviors (though see Vocate, 1994), and attempts at establishing a field of intrapersonal communication have been met with resistance (Cunningham, 1992; Hardy, 2006).

Whether intrapersonal “communication” is suitably named, this definitional problem has not otherwise been solved. There is a long-standing discussion to define communication as a general concept to underpin an emerging scientific enterprise. Over 75 years ago, (Platt 1955) asked “what do we mean, ‘communication'?”, seeking a general formulation for teaching and research in an emerging discipline. The result of this exercise was abstract and complex, because “communication” can connote a wide variety of senders, receivers, mediums and underlying features of each (Platt, 1955). Decades after Platt, (Andersen 1991) and (Motley 1991) still debated over whether communication should be so broadly defined that it is almost impossible *not* to communicate across any kind of mutual social observation (e.g., communicating one's culture with attire, one's desires by movements, etc.).

The concept of intrapersonal communication can also be found in these early debates. (Barker and Wiseman 1966) proposed a model of intrapersonal communication along an internalized vs. externalized dimension. In a subsequent exchange with (Cunningham 1992), they argued that intrapersonal processes should be part of our understanding of communication, whether we call it communication or something else (Barker and Barker, 1992). Many models in communication consider intrapersonal cognitive processes as key ingredients to our participation in communication, even in media consumption, such as the intrapersonal components proposed by Cho et al. (2009). Further evidence for this comes from the growing landscape of computational modeling in communication science in which models are explicitly formulated with underlying intrapersonal processes (e.g., Gong et al., 2023).

Here, our general aim is to make a case for what (Barker and Wiseman 1966) implied long ago: self-directed processes can be classified as a form of communication. Because it challenges the influential multiparty format, it may seem like an unintuitive and bold thesis. Nevertheless, this very concept has been adopted by many researchers across many disciplines, including communication, psychology, child development and more [Ruesch and Bateson, 1951; see discussion in Macke (2008)]. However, we will not revisit prior debates about a general definition of communication. Instead, we will demonstrate that diverse disciplines may inspire reconsidering at least some intrapersonal processes as justifiably included among other forms of communication. Importantly, the case of mindfulness presents opportunity to highlight the utility for a unified study of intrapersonal communication, given the way it is employed toward a myriad of sensations and modalities, such as affecting experience of pain or offloading verbal thoughts through perspective shifts.

In the next section, we offer a discussion of varied literature surrounding intrapersonal communication as a construct, and then share a series of empirical and theoretical observations that frame an understanding of intrapersonal communication as an adaptive category of behaviors. These will include the psychological and emotional functions of intrapersonal communication, its emergence and psychological development, and uses in day-to-day life. We end on some general theoretical reflections for this process, including how it related to concepts of consciousness and mindfulness.

## 2 Background: a varied literature

We begin with a provisional definition: intrapersonal communication is the generation and transmission of information in which sender and receiver are the same cognitive agent. This fairly general definition leaves open important discussions to eventually better define boundaries between intrapersonal communication and other phenomena, such as consciousness and cognition. Consciousness, for example, may be more specific to the feeling of experience, while intrapersonal communication focuses specifically on the information flow that may or may not be volitionally attended to. We discuss one possible framework for determining boundaries in Section 6.2.

One testament to the persistence of the *intrapersonal* communication concept is the many terms associated with it, and the theoretical frameworks it has shaped across many disciplines. In some work, a signaler and receiver contained in one individual or entity is termed *autocommunication* (Broms and Gahmberg, 1983). Autocommunication may describe individuals or organizations, and is used as a way to update the ideal state of self or “produce the information necessary to maintain itself” (Christensen, 1997, p. 200). Autocommunication was proposed by Yuri Lotman as part of a theory of general semiotics (Kull, 2015), and may be considered present even in heterocommunication, or communication between separate individuals (i.e., the traditional model of communication as social).

Research in *intrapersonal communication* takes a similar approach, but while autocommunication tends to appear in culturally specific contexts such as marketing (Christensen, 1997) or the workplace (Morsing, 2006), intrapersonal communication focuses on individual communication and relies less on institutional influences. Intrapersonal communication may also encompass a wider range of behaviors, potentially including dreaming and biofeedback training, for example (Jandt and Beaver, 1973). One major component of intrapersonal communication is imagined interactions, where individuals rehearse potential social communication. In a study by Honeycutt (2019), individuals shared anecdotes of imagined interactions they had. These individuals highlighted the use of intrapersonal communication in rehearsing highly relevant social relationships, such as romantic relationships and conflict.

Many scholars have criticized the notion of treating these processes as “communicative.” In a review and critique, Cunningham (1992) posed a summary view of intrapersonal communication as a field of study and raised several conceptual challenges to its inclusion within communication. First, it was noted that in intrapersonal communication an individual is treated as a plurality, and suggested rather that intrapersonal communication lacks “a community of at least two persons,” (p. 605) as well as other features such as the sharing or transfer of meaningful or informative messages. Second, Cunningham also criticized empirical methodologies of intrapersonal communication, and pushed back against the inclusion of inner processes, such as physiological influences. But he suggested that any externalization, such as language used to talk overtly to oneself, “[disqualifies] itself as an inner, self-contained exchange” (Cunningham, 1992, p. 608).

Cunningham's critique is clear and incisive, but these two main points of his criticism can be addressed. Consider, for example, the concern about individuals treated as a plurality. There are many studies in cognitive and neural science suggesting internal processes do have this quality. For example, binocular rivalry studies in which different images are shown separately to left and right eyes have shown that information can be available to some but not all subsystems in the brain (Lau, 2022). Relatedly, split brain research has shown the many fascinating ways that low and high level information can become disjointed internally by multiple, often non-interacting systems in the left and right hemispheres (for a review see Gazzaniga, 2005). At a higher level, separate mental mechanisms may interact across subsystems of our wider cognitive architecture (Kurzban and Aktipis, 2007). In a recent study, a computer model with simulated agents that have “multiple selves” may help explain aspects of complex decision-making (Dulberg et al., 2023). Indeed, it would be surprising if mechanisms used to deliberate with the self do not have any function, such as potentially unifying information across domains.

Consider the second of Cunningham's criticisms, that studying “internal” processes is somehow disqualifying. While certainly some modalities of self-communicative behaviors are inaccessible to current research methods, language does provide opportunities to understand how we perceive our own intrapersonal communication, such as hinting at various hidden individual differences (cf. Lupyan et al., 2023). In fact, understanding the aspects of intrapersonal communication that remain elusive will be informative to interpersonal communication itself. As we communicate with others, we are mostly aware of what we are communicating, we monitor how it may be interpreted, and how it represents us—we are not completely unaware to the influence of our own utterances (Giles, 2016). Aspects of mental traits or states can trickle into linguistic styles and other dynamics may be informative, as we will discuss in Section 6.2.

Intrapersonal communication may in fact unify several concepts that are immensely meaningful to everyday experience. These concepts have been widely discussed by philosophers of mind, who take interest in the potential division between internal (mental) and external (social, situational) processes for understanding how human experience emerges and evolves. Yoshimi (2012) summarized this philosophical debate, and offered an alternative theoretical perspective that unifies them: conscious experience is a coupled function of intrinsic (mental) dynamics and the impacts of external social and situational domains. In such debates, intrapersonal communication would be valuable to develop as a systematic empirical study. It can be integrated with this and many disciplines as a distinctive contribution of communication research which seeks to understand the exchange and influence of information across many sorts of acts of communication and many distinct kinds of parties involved. Cunningham's important criticisms provide valuable launching points for revisiting and further developing this broader area of intrapersonal communication research. From here, this range of behaviors may be better situated in the communication landscape.

The National Communication Association changed the name of “The Commission on Intrapersonal Communication Processes” to “The Communication and Social Cognition Division” in 2001 (National Communication Association, 2023). This decision framed social cognition as a dimension of broader intrapersonal communication and may have been aimed at opening new avenues of investigation that link cognition and communication. Presently, the International Communication Association does not have a clear division where research relating to intrapersonal communication could be reported. It could be argued that these disciplinary structures are meant to isolate social cognition as a specific *aspect* of communication and not necessarily a phenomenon of communication itself. Still, these decisions abandon a wider understanding of a complicated yet widely recognized inner mental ecosystem, favoring proximate description at the total expense of ultimate explanations.

There are many terms that appear best unified under intrapersonal communication, with varying descriptors across relevant literatures including autocommunication, imagined interactions, inner speech, private speech, self-talk, and more. Different terms appear more common in certain domains, such as “private speech” examined in studies of young children's intrapersonal communication (Alarcón-Rubio et al., 2014). In general terms, these various concepts can be subsumed under one notion: our personal thoughts can have the appearance of internalized communication. They may be overt, subdued, or covert vocalizations produced in isolation, written forms such as journaling and thought-listing, and more. As such, intrapersonal communication will be defined here as communicative behaviors or processes where the sender and receiver are contained within an individual (cf. Ruesch and Bateson, 1951).

A casual search across various literatures yields many instances of these concepts. While a systematic review permits certain search criteria and hypotheses in advance, our initial survey of this concept across various literatures led to clear challenges to this approach. For example, the very terms associated with intrapersonal communication, its effects or covariates and so on, are all quite varied across these literatures. For this reason, we engaged in an exploration of related concepts across these literatures and organized these observations in the present review. Indeed, with these findings in hand, it may be possible to conduct a more systematic inquiry scanning the journals and topics summarized in Table 1. Despite these wide ranging concepts, they rarely appear in communication journals. A search in the Web of Science, for example, revealed that communication journals rarely published work related to concepts like inner speech, private speech, or self-talk, despite their conceptual relatedness to intrapersonal communication (Table 1). Here, we have selected a few of the concepts that appear in the literature and have clear connections to communication given their linguistic nature—certainly, several other concepts that may be considered intrapersonal communication could show interesting patterns in where they are or are not published. However, we suspect if even self-talk and inner speech are underrepresented in the field of communication, it is unlikely that other concepts such as mindfulness would be more prominently featured.

**Table 1:** Top 10 journal topics per search term.

**Ranking - most common publication areas**	**Search term**				
	**Intrapersonal communication**	**Inner speech**	**Private speech**	**Self-talk**
1st	Education educational research	Neurosciences	Psychology developmental	Sport sciences
2nd	Communication	Psychology experimental	Education educational research	Psychology applied
3rd	Psychology multidisciplinary	Psychiatry	Linguistics	Hospitality leisure sport tourism
4th	Public environmental occupational health	Psychology multidisciplinary	Psychology experimental	Psychology
5th	Psychology clinical	Psychology	Psychology multidisciplinary	Psychology multidisciplinary
6th	Social sciences interdisciplinary	Linguistics	Language linguistics	Psychology clinical
7th	Health care sciences services	Philosophy	Psychology educational	Education educational research
8th	Family studies	Psychology developmental	Psychology	Psychology experimental
9th	Psychology social	Education educational research	Rehabilitation	Psychiatry ^*^ (tied with psychology experimental)
10th	Health policy services	Clinical neurology	Law	Psychology social

The top 10 publication areas represented by articles listed on the Web of Science as of April 2024, based on search term. While communication appears as a top publication category for intrapersonal communication which may be simply because the word “communication” is present—inner speech, private speech, and self-talk all do not show communication as a main topic at all. Even for intrapersonal communication, communication is secondary to educational research.

Regardless of these varied notions across disciplines, so many potential manifestations of intrapersonal communication suggest they could play an important role in our social cognition, communication, and beyond. Here, we will cover a wide range of intrapersonal communication concepts, most often studied independently from one another, across different disciplines, and without much consideration of the *communication* aspects in particular. We will explore different contributions to intrapersonal communication from these various fields, in an effort to highlight how a unified study could combine existing work to most effectively build on further investigations. Inspired by the broad array of disciplines in Table 1, this review develops an interdisciplinary approach, integrating research from multiple fields to gain insight into any such unified phenomenon. In the following section, we first suggest that evolutionary and developmental evidence supports the idea of intrapersonal communication as a valuable adaptation, one that may even guide our understanding of human consciousness.

## 3 Functions of intrapersonal communication

Several high-level cognitive processes are likely important to human functioning, such as metacognition (our ability to think about thinking) and our capacity for theory of mind (to reason about the mental states of others) (Baron-Cohen, 1999). It may be that intrapersonal communication itself serves related adaptive functions. What use would we have for intrapersonal communication processes? In this section, we present an evolutionary inspired survey on why intrapersonal communication is a viable concept by considering its potential functions.

### 3.1 Information search

One highly relevant domain of study in cognitive science is internal foraging or search (Todd and Hills, 2020). We engage in mental foraging routinely, looking for concepts or ideas to formulate our thoughts and communications. For instance, if asked to list all the animals one can in a limited amount of time, individuals may internally search across various topics, from farm animals to a new category such as common household pets. When people do these cognitive tasks, their behavior tends to have many statistical features of a physical foraging process (Todd and Hills, 2020). Just as we engage in exploitation of resource rich environmental patches, and explore when seeking more resources (such as when a patch has been depleted), we engage in similar patterns exploring mental space. Given these similarities between external and internal foraging, self-awareness may have its origins as a mechanism for distinguishing mental and external foraging. Hills and Butterfill (2015) proposed that it was adaptively advantageous to distinguish resource foraging thoughts from the real external environment, resulting in the evolution of self-awareness or “the capacity that allows adult humans to mentally represent to become aware of their protracted existence across subjective time” [quoted from Wheeler et al. (1997) in Markowitsch (2003), p. 181]. Tooby and Cosmides (2001) took a similar approach to the construction of fictional worlds where reality and fiction must be effectively distinguished, and people are able to move effortlessly between such representations with minimal confusion.

The possibilities introduced by intrapersonal communication would be limited only by the imagination of the self-communicator. Creating a kind of internalized fiction allows imaginative possibilities to be explored and may benefit the maintenance and development of mental processes much like exercise works the body, or choosing a habitat works the external world (Tooby and Cosmides, 2001). This capacity, rather than being explicitly socially oriented, benefits the individual in all domains in which decisions shape outcome payoffs. With greater abilities to volitionally forage creatively in the mind, such as through language, the possibility space can be quite large.

### 3.2 Imagined interactions and versions of the self

The adaptive benefits of imagined interactions (IIs) may be easy to recognize. These rehearsed social engagements could constitute a major aspect of intrapersonal communication. For example, imagined interactions allow for the processing of norm violations (Berkos et al., 2001). In one study, students encountered one of three instances of teachers violating norms (i.e., being incompetent, offensive, or indolent) and were asked to report IIs with the target teacher as well as the likelihood they would actually engage in said interaction. First, when asked about general use of IIs to cope with norm violations, students significantly used IIs relative to a theoretical mean. When using IIs to process the norm violations presented in the study, they used them in the place of real-life interactions. As such, the imagination can facilitate both exploration and avoidance of possible interactions.

This socially guided processing can be applied to the self as well. IIs may also involve different versions of the self. The dialogical self, proposed by Hermans (2002), involves internal dialogues or conversations, considering models (such as real-life social partners, or fictional or distant others) to shape one's internal interlocutors. The dialogical self-perspective is necessarily social in nature, and generally extends to representations of society, culture, and history in seemingly infinite internal selves (Hermans, 2002, 2003). The interlocutors within the dialogical self may include specific roles, such as Faithful Friend, Ambivalent Parent, Proud Rival, and Calm Optimist (Puchalska-Wasyl, 2015). Such techniques of separating out aspects of the self have proven valuable in the clinical realm, supporting the notion there may be distinct functions for intrapersonal communication, particularly self-talk (Schwartz, 2013).

Self-talk also appears to feature a variety of interpersonal styles employed under different affective states (Lefebvre et al., 2022). Some self-talk may serve self-management, self-critical, self-managing, and self-assessing functions (Brinthaupt et al., 2015). It may serve performance as well, such as motivational and instructional self-talk, considered especially in sports psychology (Hatzigeorgiadis et al., 2004) and addressed further in Section 5.2.

Connections of self-talk to clinical psychology go back to Freud (Lapsley and Stey, 2011). The ego includes unconscious monitoring and suppression of the self, which logically extends to self-deception. Von Hippel and Trivers (2011) suggested that self-deception may facilitate social advancement by allowing for deceptive self-inflation. Similarly, Goffman (2004) posed the self as performative, where we engage in behaviors specifically to modulate others' perception of us. Intrusive thoughts provide an interesting example of dissonance within the self—while we may recognize these thoughts as generated from ourselves, we do not necessarily volitionally send these thoughts out, and we often do not wish to identify with them. However, suppression is associated with increased intrusiveness, and leads to increased levels of distress (Marcks and Woods, 2005). Cognitive dissonance provides a similar example of tension within the self, where we make a change to our beliefs or behaviors to resolve the discomfort of such inner conflict (Festinger, 1957). The existence of self-deception, intrusive thoughts, and cognitive dissonance regulation strategies all imply the existence of a disjointed self and a suite of mechanisms that helps balance different streams of information about the self.

### 3.3 Neural functions

Neurological data could potentially provide insights into psychological functions of intrapersonal communication. In neuroimaging, the network of activity occurring when not performing a task may be associated with a default state of mind-wandering (Bar et al., 2007). This default mode network (DMN) may work in conjunction with a frontal-parietal network (FPN) to generate our streams-of-consciousness (Smallwood et al., 2012). As suggested by Smallwood et al. (2012), this pairing of the DMN with a control network such as the FPN permits spontaneous trains of thought to occur. The DMN may also connect thoughts to different mental health conditions. Major depressive disorder, particularly the tendency toward rumination and brooding, is associated with patterns of higher rest-state connectivity (i.e., the DMN: Berman et al., 2011). Understanding the relationship between the DMN activations and intrapersonal communication activities, content, and outcomes may further reveal functions for this internal process. We can consider these internal brain dynamics as a kind of intrinsic intrapersonal process (Stacks and Andersen, 1989).

Verbal intrapersonal communication may link to other social and emotional neural systems. For example, the labeling of emotional faces has been shown to reduce activation in the amygdala and other parts of the limbic system, suggesting diminished emotional reactivity (Lieberman et al., 2007). Different language-associated regions of the brain may also reflect the different roles of sender and receiver within one individual. As noted by Gibson and Foster (2007), the left frontal cortex tends to be associated with language production, while regions in the left temporal cortex are often associated with comprehension and monitoring of language and self-talk. Research findings with schizophrenic patients who experience auditory verbal hallucinations are consistent with these brain activity patterns. Thoughts also do not necessarily rely on language as evidenced through neuroimaging of individuals with global aphasia who have limited verbal skills (Fedorenko and Varley, 2016).

Our understanding of how different intrapersonal communication methods and modalities operate may also be influenced by what model of brain function is applied. In functionally specialized models of cognition, domain-specificity may support the notion of independent internal entities that could benefit from intrapersonal communication and regulation. However, many of the mechanisms likely to enable intrapersonal communication require higher-level cognitive abilities that integrate and organize multiple lower-level mechanisms. Barrett (2005) proposed an enzymatic computation model where outputs from lower-level systems can be pooled, and then interpreted in specific ways if they match the input criteria for a given “cogzyme,” or cognitive device that operates using a “lock-and-key” style of computation, comparable to enzymes. By this model, there is a dynamic co-existence between functional mechanisms and integrative connections between them, giving rise to a powerfully flexible, and specialized architecture. These cogzymes can be potentially activated by stimuli that satisfy input conditions even if somewhat different from the set of features the device is designed to process (also described as a difference between actual and proper domains of a mechanism) (Sperber, 1994). This plasticity could be by design as a strategy to manage different kinds of perceptual errors (e.g., snake detectors have liberal criteria creating a greater chance of a false alarm than a miss). Intrapersonal communication may be designed to generate such keys to exploit the input conditions of different cognitive mechanisms, allowing the mind to practice responses beyond what is available in the immediate external environment. Given that intrapersonal communication engages imagination, such as mentally traveling through time, space, and abstract possibilities, it may then model real-like experience to tap into mechanisms like social bonding, reward and punishment, and so on.

### 3.4 Cross-cultural variation

When considering the functions of intrapersonal communication, it is also valuable to recognize how it may manifest variably across cultures. A study using Brinthaupt's US-based Self-Talk Scale (Brinthaupt and Dove, 2012) with a Chinese sample found reliability in self-talk being employed for self-criticism, self-reinforcement, self-management, and social assessment (Ren et al., 2016). While the functions and characteristics of self-talk may share commonalities across cultures, it will likely vary as a function of cultural norms of expression. In a study comparing the IIs used by young adults in the US, Thailand, and Japan, some differences were found. First, Americans were found to exhibit more self-dominance, or dominating of conversations in IIs (McCann and Honeycutt, 2006). Valence, frequency, and variety of IIs was also found to vary across these three cultures. However, it is important to note that each of these samples were comprised primarily of urban student populations. Variation in imagined interactions, and likely other self-talk phenomena, should be expected both between and within cultures as the utility of self-talk will be adaptive to the needs of the individual in the contexts they occupy. Even in cases of psychosis-related acoustic hallucinations, cross-cultural differences have been found (Luhrmann et al., 2015), suggesting that a range of intrapersonal communication behaviors and processes should be examined with a wider cultural lens.

Different forms of intrapersonal communication appear across cultures as well. For example, the Shuar of the Amazon sing *anent*, which are used with the intention of influencing outcomes in the world in some form (Rubenstein, 2012). While anent can be shared with others (with certain limitations), they need not be explicitly communicative toward others in nature and are not understood by those who have not been taught that specific anent. Prayer, a cross-culturally robust phenomenon, may also have connections to intrapersonal communication. While prayer may include the implication of an external communication partner, it generally does not play out with the same turn-taking dynamics of interpersonal communication. No difference in stress levels was found in a study comparing prayer and self-talk, suggesting both may serve similar intrapersonal functions (Belding et al., 2010).

Cultural norms likely shape the strategies for communicating with oneself in ways that may impact functional outcomes. An important trajectory for intrapersonal communication studies is in recognizing what variation is present, as well as where universalities may hint at deeper functions of self-directed communications. Some variation is only just being investigated, even in how different individuals experience their own thoughts (such as a reported lack of inner voice—see Nedergaard and Lupyan, 2024; see also Fernyhough and Borghi, 2023 for some discussion). Through necessary cross-cultural work, a deeper understanding of the possibility space of our self-communications may be possible.

### 3.5 Summary

The functions of intrapersonal communication can be consciously engaged, such as searching our memory for information or ideas. It can also be subtle, such as the intrinsic dynamics of the brain while it is not pre-occupied with a task. Cultural variation suggests that self-talk may be a blend of deliberate self-regulation along with possibly unconscious influences of one's cultural context. The functions of intrapersonal communication likely have both conscious and unconscious elements, allowing one to adapt to ecological and social environments. We will consider how these conscious or unconscious elements may help to categorize intrapersonal communication in Section 6.2.

## 4 Development of intrapersonal communication

If the functions described above characterize a core psychological contribution of intrapersonal communication, then we may see them emerge systematically during childhood learning, including in the development of various modes of intrapersonal communication.

### 4.1 Early development

At birth, intrapersonal communication does not have verbal language as a medium for expression. However, as skills build early in life, different vehicles of intrapersonal communication may unfold. A study with preverbal children aged 14, 16, and 18 months of age found self-regulatory private gestures used during play activity, possibly representing precursors to private speech (Basilio and Rodríguez, 2017). Even newborn infants engage in imitation of body movements, that may be reflective of an initial implicit consciousness enabling subsequent explicit consciousness-related behaviors (Lewis, 2003), such as intrapersonal communication or self-talk. Mechanisms such as imitation or recognition of the self in mirrors show the capacity for parsing the self from others.

Intrapersonal communication in children typically transforms into private speech (Stanley, 2011), which initially involves vocalizing verbally and overtly to oneself with no intention for an external audience to receive any messages. Infants as young as 5-months-old may also be engaging in deliberate vocal play when alone. In a study by Shimada (2012), infants vocalized for longer durations when left alone by the parent(s), and used significantly more acoustic phrase repetitions. Interestingly, in a condition where the infant vocalizations were amplified in real-time, infants prolonged their vocalizations, altogether suggesting the goal was specifically vocal play rather than elicitation of parental attention (cf. Oller et al., 2013).

### 4.2 Debate on developmental trajectories

The nature of early self-talk such as private speech has provoked differing perspectives on the connections to social communication. A Vygotskian perspective on development suggests that self-talk starts in the social realm, moving to internal mental processes afterwards to enable self-regulation (Winsler, 2003). This may be reflected in the tendency for overt (external) private speech to accelerate starting around 3 years of age before withdrawing internally around age 7 (Stanley, 2011). In the transition to internalized intrapersonal communication, partially internalized private speech occurs, with whispered or mouthed speech patterns. This partially internalized speech may have a self-regulatory as opposed to a social function (Alarcón-Rubio et al., 2014).

However, variation in the overtness of private speech may be dependent on context. A recent study using three separate tasks involving delayed sequential memory, selective attention, and a Tower of London test, found differing degrees of overt speech based on the task (Doebel and Munakata, 2022). For example, age effects (from 5- to 7-year-olds) were only found for frequency of self-directed speech on the selective attention task. A Piagetian perspective, in contrast to a Vygotskian one, argues that children rarely take a social perspective, instead making use of egocentric speech with social speech emerging as a product of developing logical thought (Stanley, 2011). This suggests equating intrapersonal communication to social cognition in communication may be premature or incomplete.

Interestingly, despite burgeoning use of private speech in the preschool years, it is unclear how or when awareness of internal thoughts or streams-of-consciousness develops in childhood. In a task evaluating willingness to attribute active mental states to others, or even themselves, younger children appear to perceive waiting periods as moments where no thoughts are happening (Flavell et al., 1993). In a task eliciting volitional streams-of-consciousness, kindergartners struggled with production compared to fifth graders (Kipp and Pope, 1997). However, pretend play, or pretense, appears during the second year of life, if not earlier, in the form of pretend gestures (Fein, 1981), showcasing that regardless of metacognitively generated self-reports, young children are able to generate fictionalized explorations independently in communication with the self. Evidence suggests the ability to attribute mental states to others emerges by 3–4 years of age or quite earlier (e.g., 13 months) when tasks are not based on verbal measures (Surian et al., 2007), and as such it may be more precise to consider intrapersonal communication as early emerging yet variable as various cognitive capacities unfold (Lewis, 2003).

Understanding variation in intrapersonal communication capacity, for example in volitional control due to self-regulation abilities, may inform future discussion of what counts as consciousness, what distinguishes human consciousness from potential forms in other species, and how consciousness changes throughout the human lifespan.

### 4.3 Emergence of emotional and other functions

Both Vygotsky and Piaget considered self-regulation as a key mechanism relating to the self. While both considered self-regulation from an intellectual perspective, Piaget also highlighted the self- regulation of emotion (Fox and Riconscente, 2008). Research on the private speech of children used in a frustration task showed differences in self-talk usage in relation to emotional valence and regulation strategy (Day and Smith, 2013). For example, even when controlling for regulation strategies, negatively valenced task-relevant private speech, along with higher levels of social speech, predicted higher levels of sadness. Task-relevant self-talk appears to have both positive and negative effects on task performance, depending on various factors.

Greater frequency of overt task-relevant speech may be associated with lower inhibitory control and executive function issues (Thibodeaux et al., 2019). However, task- relevant private speech may be beneficial in cases where the task is neither too simple nor too difficult for the child (Fernyhough and Fradley, 2005). In another study examining the task-relevance of private speech, Mulvihill et al. (2021) found in both a Duplo construction task (i.e., replicating a specific Lego^®^ structure) and a card sort task a high frequency of performance-related content, such as self-instruction, attention focusing, and observational statements. Task performance was particularly influenced negatively by task-irrelevant content. Forethought content, such as motivational language or future planning, was associated with improvements in task performance in some cases.

### 4.4 Family and social systems

Given the social nature of language learning, it is unsurprising that the nature of children's self-talk will also be influenced by experiences with parents and other significant individuals in early life. Overall, children who perceive the people in their lives as speaking to them positively use more positive self-talk and less negative self-talk, with the opposite pattern emerging when they perceive others using more negative talk toward them, although sex differences may suggest differing social pressures based on gender norms (Burnett, 1994).

Similarities have also been found in child and parent narratives about traumatic events, even when sampled separately from one another (Alisic et al., 2016). These similarities include length/elaboration, rates of anxiety words, and rates of cognitive words. The makeup of a family may also influence self-talk: the frequency of self-talk, especially self-critical talk, was found to be higher for adults who grew up as only children compared to having siblings (Brinthaupt and Dove, 2012). Interestingly, this study also reported a higher frequency of self-talk in adults who had imaginary companions as children. This self-talk also included significantly more self-reinforcing and self-managing self-talk than those who did not have imaginary companions growing up, and suggests positive benefits to imagination that may play a functional role for intrapersonal communication.

### 4.5 Summary

Developmental research on intrapersonal communication supports the idea that it is self-regulatory in nature. But there remain a number of intriguing questions about the developmental ordering and origins of the process, as the earliest speech can occur privately, but with potential systematic effects of the child's social environment.

## 5 Intrapersonal communication in everyday life

As described above, various functions of intrapersonal communication can be identified in the experiences and behaviors of young children. As a broad repertoire for engaging the self, it should also be flexible under various factors and conditions. This flexibility may derive, in part, from the capacity for intrapersonal communication to manifest itself in both conscious and unconscious processes. Below we cover some exemplars in day-to-day life where this flexibility is present.

### 5.1 Cognitive factors and distancing

Self-talk relies on language production and is thus likely to be constrained by priming that directs the flow of thoughts. This mental process can sometimes be very rapid and unconscious. For example, syntactic priming research demonstrates how the processing of one utterance's form influences the processing of subsequent utterances (Pickering and Branigan, 1999). The framing of thoughts in different times, spaces, and perspectives also has implications for the construction of self-talk. In a review by Trope and Liberman (2010), times, spaces, and perspectives that are considered more distal from the here and now and one's identity led to more abstract mental construals. Observations regarding framing are central in construal-level theory. Increased abstraction appears to influence self-talk depending on context. One way psychological distance is achieved is through the use of first- vs. second- or third-person pronouns, and this may impact performance on tasks. For example, when asked to give advice after imagining a specific scenario, individuals who were primed to give advice in the second-person (i.e., using “you” instead of “I”) performed better on an anagram task (Dolcos and Albarracin, 2014). Distanced self-talk may also enable more rational thought, with a third-person perspective of the self leading to better gains in strategic games (Gainsburg and Kross, 2020).

There is evidence that this distancing factor relates to aspects of health-related feelings and outcomes (Kross et al., 2017; Furman et al., 2020; Oliver et al., 2016; Orvell et al., 2021). For example, third-person self-talk may be a low-effort technique for emotion regulation, with reduced event-related potentials (ERPs) in a marker associated with self-referential emotional reactivity despite no enhancement in an ERP marker of cognitive control (Moser et al., 2017). Third-person self-talk also appears to more strongly influence self-conceptualization than adopting the perspective of a close friend or thinking of the self in first-person, leading to more abstract language (Gainsburg and Kross, 2020).

Distanced self-talk can also occur beyond personal pronoun anchoring. In work on decentering, de-identifying the self with certain aspects of an experience is used in some mental health interventions. Rather than construing oneself as “being sad,” for example, they may instead “have sadness,” minimizing the hold the feelings have on the individual (Bernstein et al., 2019). Temporal framing of thoughts may trend toward the past being associated with depression and the future being associated with anxiety (Pomerantz and Rose, 2014). Circumstances may also matter, such as the life-altering effects of COVID-19, and may shift how temporal framing influences future thoughts (Bainbridge and Dale, 2023). Additionally, the effectiveness of temporal framing as a deliberate intervention may depend case-by-case based on individuals' attachment styles, which could be proxies for baseline tendencies toward proximal or distal psychological distances (Wang et al., 2012). Low-avoidant individuals show less negative emotion and more positive emotion using psychological distancing after reading a threat-inducing scenario, while high-avoidant individuals (i.e., those with psychologically distanced tendencies) showed no meaningful benefits. While avoidance may be associated with a tendency for further psychological distances, anxiety may lead to closer than average distancing. Both low and high anxiety participants were able to lessen negative emotion through distancing, although only those with low anxiety increased positive emotion through distancing.

### 5.2 Contextual variation

Distancing is only one way self-talk can be employed to guide outcomes. As we have seen with research on private speech in children, task-related language can be beneficial, depending on varying details of the context. In the domain of sports psychology research, evidence suggests instructional self-talk may increase precision on tasks, while motivational self-talk may benefit strength and endurance (Hatzigeorgiadis et al., 2004). Motivational self-talk of athletes using a stationary exercise bicycle was shown to reduce time to exhaustion as well as reducing ratings of perceived exertion when partway through this physically exerting activity, permitting greater endurance (Blanchfield et al., 2014). This suggests self-talk is guided by context. Being in an autonomy-supportive environment (e.g., giving rationale, validating the participant's perspective) lead to greater use of positive emotion words, fewer negative words, and fewer first-person references in a think-aloud task compared to being in a more controlled environment (Oliver et al., 2008).

Our streams-of-consciousness may also be constrained by cognitive load. For example, when exposed to stimuli at a faster rate, or with greater short-term memory loads, task-irrelevant thinking and visual imagery are lower, hinting at limitations to mental foraging when cognition is tied up elsewhere (Antrobus et al., 1966). The common experience of mind-wandering may relate to intrapersonal communication, and it is also influenced by working-memory load (Soemer and Schiefele, 2020). Tip-of-the-tongue phenomenon, where problems accessing a specific word is subjectively felt, also presents an example of where streams-of-consciousness may become disrupted, particularly when verbally guided. Tip-of-the-tongue has signatures of greater cognitive load, with greater pupil dilation during failures to retrieve a tip-of-the-tongue answer (Ryals et al., 2021). While mind-wandering might not be inherently bad, it seems to be best served when other processing is not necessary. Similarly, when other tasks demand cognitive power, mind-wandering may be particularly disruptive (see also Jones and Langford, 1987; Soemer and Schiefele, 2020).

Intriguingly, the growing practice of mindfulness may relate to intrapersonal communication. Mindfulness does not necessitate language, and indeed often recommends avoidance of verbal mind-wandering by transferring focus elsewhere (Creswell, 2017). Mindfulness often applies attention to in-the-moment sensations and experiences (both internal and external), which can include the observation of verbal thoughts but aims to anchor against the streams-of-consciousness. The focus on physical sensations in mindfulness practices appears as one way to shift intrapersonal communication from uncontrolled verbal streams to interoceptive or exteroceptive (i.e., external) awareness. Interoceptive awareness, or the awareness and evaluation of the body's physiological workings and state, appears higher for those who are also high in dispositional mindfulness (Hanley et al., 2017). It may therefore be possible to adapt one's self-communicative mindfulness willfully. Deliberately observing physical sensations can help tune dispositional mindfulness, which in turn can improve benefits of using other modalities of intrapersonal communication, such as writing to the self (Quaglia et al., 2016).

### 5.3 Summary

As suggested by the function and development of intrapersonal communication, cognitive features also reveal a variety of mechanisms that may support it. An unconscious process like linguistic priming may shift and guide intrapersonal thinking, while active and deliberate engagement may help people shape their own internal processes.

## 6 Discussion

Intrapersonal communication represents a collection of rich phenomena, developing systematically, varying cross-culturally, and subject to conscious and unconscious cognitive processes. Some research illustrates functionality in intrapersonal communication, which can be valuable when considering how these behaviors may be adaptive (and indeed, future work may consider these functions when exploring non-human animal behaviors that may serve similar purposes). Information search and mental foraging could reflect adaptations for navigating complex external environments. Social mechanisms moved internal might function to manage aspects of the self and interface with external norms, such as through imagined interactions. When inner selves come in conflict, cognitive dissonance and self-deception may emerge. Neuroimaging work might provide insights into how intrapersonal communication can be neurally implemented, with some promise in looking at the default mode network, as well as emotion and language relevant regions. Current and future cross-cultural work will provide important insight into what should be considered intrapersonal communication, and where possible boundaries should be drawn.

The early emergence of private speech, and even private gestures in preverbal children, suggests the importance of intrapersonal communication during development. Intrapersonal communication connects to task performance and emotional regulation in children. As work in the development of intrapersonal communication diversifies across modalities, interesting implications for future mental health and behavioral outcomes may emerge. Performance and endurance in sports and other physical activities can be influenced by self-talk and other mindfulness practices, but how does our adult intrapersonal communication landscape manifest or become constrained by our early-life experiences?

We argue that the appearance of so many behaviors and capacities resembling intrapersonal communication suggests it is a viable concept to be studied as part of communication science. But to bring further coherence to the concept, there are at least two important next steps in this enterprise. First, the approach is theoretical in nature, such as how we situate intrapersonal among other forms of communication, and systematically relate these concepts across academic disciplines. The second step is methodological, integrating research into a well-understood toolkit to measure intrapersonal communication. We consider these two puzzles of integration in the next sections.

### 6.1 Integrating theory

Theoretical approaches to communication such as information theory (Shannon, 1948) provide an initial perspective on how intrapersonal communication might operate. In some of our self-directed communication, we are deliberately sending and receiving the messages of our thoughts. For example, imagining social interactions or deliberating with yourself to conclude your opinion on a topic may appear similar to interpersonal communication. There may even be strong modality effects, such as imagining the tone of your inner voice, as it tells you how you might feel about current events. Perhaps the noise in this internal channel is made up of other voices or ideas popping up, interrupting your thoughts. Actively attending to thoughts allows one to receive some potentially coherent message, allowing for desired behavioral or information-state changes. Some intrapersonal communication may involve less awareness, however. To better understand the variation of behaviors that may be classified as self-communicative, researchers should consider conscious involvement in various aspects of the sending and receiving of messages with the self. While total lack of conscious involvement may constitute mere cognition, active engagement in sending, receiving, or completing a full communication chain could be distinct enough to qualify as intrapersonal communication.

Theorists should also consider why and how intrapersonal communication is even possible. This includes considerations from the previous sections, as well as how novel information may be possible to generate and communicate with the self. Under the stochastic generation of information, it is possible to derive novel combinations of ideas, processing, and reevaluation (see Coutanche et al., 2020 for a review). But the level by which the self-communicating entities are defined also matter. These levels can be defined at the micro scale (such as neuronal communication) but also, at least theoretically, between multiple “selves.” While communication-like processes do occur between individual neurons, this level may not be especially meaningful on its own for representations of self-hood or other major cognitive or behavioral outcomes. Instead, at a higher level, it is likely more aggregate, emergent cognitive phenomena that underlie this capacity to “inform the self” through internal dynamics.

Future models will be able to present this capacity across levels of entities, such that neuron to neuron, brain region to region, self-representation to alternate selves, and even interpersonal systems, may all be linked. Questions of this nature may initially be beyond the scope of intrapersonal communication as a discipline of empirical study, but this does not necessarily rule out multiple levels or conceptions of selves that communicate intrapersonally. Rather, certain levels should be considered the primary thresholds necessary to constitute communication with the self (as opposed to being the mere raw ingredients affording intrapersonal communication). Theories of intrapersonal communication thus would benefit from considering theories of self and identity (McAdams and Cox, 2010) to determine meaningful categorical boundaries.

### 6.2 Active/inactive sender-receiver model

To better understand how different modes of intrapersonal communication might operate, an adapted model of communication should clarify how a sender and receiver exist within the self. Here, we propose a preliminary framework that enables the self to be engaged as sender and receiver. As mentioned earlier, the sending and receiving can be differentially conscious or “active,” in that the process may be one involving active awareness and effort. In some situations, we become particularly tuned into signals we observe within, taking on a more actively engaged role as the receiver to these messages. Other times, we deliberately formulate signals of information with the intention to have influence on the self—for example, through journaling or production of overt speech. These sender and receiver roles constrain how intrapersonal communication influences behavior or reveals inner psychological or mental states. Similar approaches to traditional, interpersonal communication have been explored to understand sender- vs. receiver-focused perspectives (Andersen, 1991). This includes exploring instances where the sender may be intentionally or unintentionally sending signals, or a receiver being receptive, incidentally receptive, or non-receptive. Is communication dependent on someone receiving an intentionally sent message (e.g., Scott-Phillips, 2008)? If so, it suggests intrapersonal communication should also be considered communication. Examples of behaviors that represent different self-directed sender and receiver activations are presented in Table 2.

**Table 2:** A matrix of self-communicative behaviors where the individual may take active or inactive sender and receiver roles.

		**Receiver**	
		**Active**	**Inactive**
**Sender**	**Active**	Imagined interactions, simulations, conscious sensory imagery (including visualization, aural imagery, interoception, etc.), overt self-talk (e.g., spoken, written), lucid dreaming	Spontaneous thoughts that are not appraised or attended to (but one could become aware of by activating the receiver role)
	**Inactive**	Dream recall, hallucinations, intrusive thoughts, emotional appraisal	Dreams not recalled, cognition, intangible thoughts such as implicit associations, biases, or unattended emotional responses

This matrix is analogous to figure 1 in Andersen (1991) explaining communication that is intentionally vs. unintentionally transmitted or successfully vs. unsuccessfully received. Like interpersonal communication, intrapersonal communication may vary in nuanced ways based on the effect it can have on the sender and/or receiver contained within the singular individual.

Active sender/active receiver represents the most involved model, where communication within the self is explicit and intentional. Imagined interactions represent a fairly even distribution of focus as the sender and receiver, as the interaction plays out dynamically with consideration of potential interpretations a receiver of the signal may have, as well as how this then feeds back into the subsequent turn of the original sender. Overt intrapersonal communication, whether spoken or typed, also likely constitutes an active sender and active receiver model, as the act of making intrapersonal communication tangible forces the self to receive the message.

While the self can still be influenced by the behaviors listed as active sender/inactive receiver, the role of receiver is inactive and lucid interpretation is not engaged. This category may be the least distinct, as the act of sending a message with awareness likely activates the receiving role more so than other inactive positionings. However, thoughts can often be generated without engaging reappraisal, leaving the role of receiver limited as it is not then taking a subsequent turn as sender in a hypothetical internal interaction. Indeed, one may become active as receiver after the fact, becoming retroactively aware of a thought that had already been generated. While the matrix in Table 2 represents a simplified sender-receiver model, the feedback relationship between partners in traditional, dyadic communication interactions is likely to play out in intrapersonal communication as well. If we indeed are able to influence ourselves via intrapersonal communication, the model is interactive.

In some cases, one may deliberately shift these roles into active positions. For example, mindfulness meditation draws on existing active signals of sensation within the body and activate the sender role by using thought to shift the experience of those sensations. Other strategies of mindfulness may activate the receiver role, such as by calling attention to sensations that have been otherwise undetected—vibration sensations in the hands or feet, for example. Varying the activation of these sender and receiver roles may provide different benefits based on the functions of an intrapersonal communication act. Mindfulness can also exploit other modalities—generating visualizations such as rivers or dissipating clouds to observe the flow or releasing of thoughts. Of course, this also includes self-talk, such as assessing and discussing our pain with ourselves (cf. cognitive appraisal: Ramírez-Maestre et al., 2008).

While most of the literature connecting to intrapersonal communication focuses on self-talk, better integration with the study of mindfulness is a valuable first step toward clarifying the boundaries of our self-directed communication and factoring in the richer possibilities multiple modalities provide. Indeed, the preliminary taxonomy in Table 2 may suggest ways of organizing different sorts of cultural experiences around mindfulness. Prayer, for example, can represent an active-sender model while opening dialogue or reflection with objects of religious experience or belief. This activity has cognitive implications. For example, Schille-Hudson et al. (2024) investigated five cultures in their prayer experiences and found that increased prayer engagement altered various cognitive, perceptual and other experiential reports. This sort of work suggests that mindfulness-related activities has broad applications in day-to-day activities across cultures. Other cultural practices invoke specialized institutions or roles. Hove et al. (2015) showed that an expert Shaman experience may involve a distinct perceptual *de*coupling from external stimulation through active focus with a rhythmic stimulus. This suggests a relatively active-recipient form that has as its attentional focus a single source of stimulus energy.

The framework in Table 2 offers a way of conceptualizing mindfulness and related experiences as a kind of intrapersonal communication where knowledge of the self (or *through* the self) has differential active/inactive forms. Overall, mindfulness may work to shift or enhance the level of active engagement in intrapersonal communication, and future work could directly apply mindfulness interventions in varying modalities or to target varying information streams within the self to assess where self-communication might be more or less effective. For example, mindfulness has shown some promise for regulating mind wandering across several studies including both behavioral and physiological methodologies (Feruglio et al., 2021). Mindfulness could be particularly valuable as a method for shifting information across the framework's quadrants (e.g., from inactive sender and receiver to active sender and receiver). Previous perspectives on mindfulness have noted its value toward self-processing and phenomenological connection, contributing toward the maintenance of one's self-schema (Vago and Silbersweig, 2012). Dispositional mindfulness has been shown to boost psychological wellbeing through enhancing self-concept clarity (Hanley and Garland, 2017). Mindfulness therefore may provide important tuning to enable intrapersonal communication to occur successfully.

Just as strategies can be employed to make *inter*personal communication clearer (i.e., reduce entropy by providing the information necessary in a minimally noisy channel), mindfulness may be a primary methodology for doing the same with endogenous signals. It is worth noting that intrapersonal communication does not guarantee successful self-regulation, just as interpersonal communication is not guaranteed to achieve either the sender's or receiver's communicative goals. However, engaging actively in the sender and receiver roles likely provides more potential control over the self compared to not. Future work should explore the role of active/inactive engagement as the sender and receiver of internal messages, as well as how other features such as modality and vividness of the messages can impact the outcomes of our intrapersonal communication behaviors.

### 6.3 Integrating methodologies

**Figure 1:**
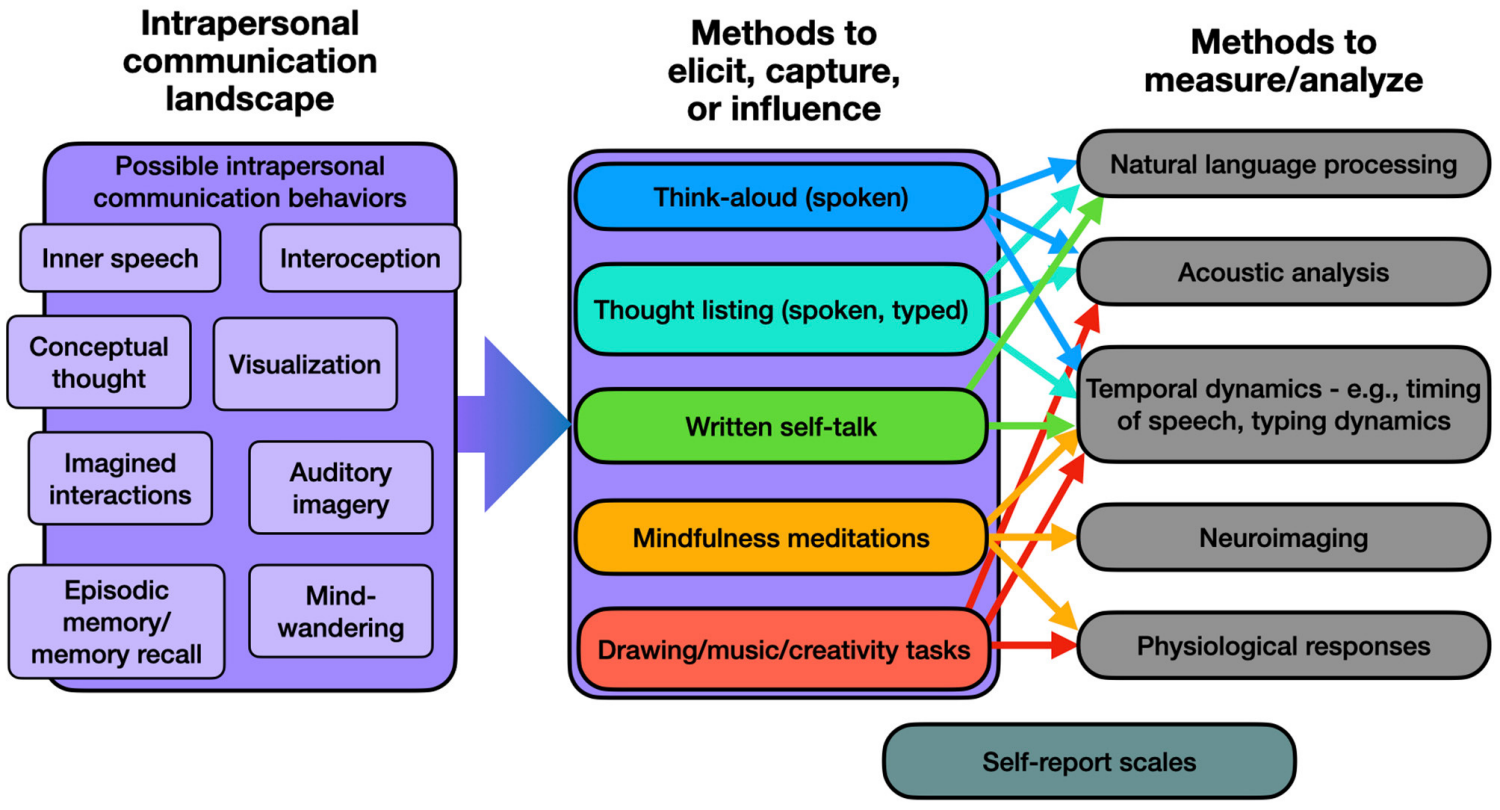
A rough portrait of a methodological approach to intrapersonal communication. We likely self-communicate through a myriad of modalities, which could be elicited or collected in various formats. From there, methodological options for analysis of these communication acts may be applied, sometimes in multiple manners such as the combination of natural language processing with analyses of temporal dynamics.

To assess all these underlying factors, various methodologies can be integrated into a general study of intrapersonal communication. One of Cunningham's (1992) prominent critiques regarded the difficulty in devising methodologies to study such internal processes. But there are techniques available for such an enterprise, even considering the rich array of studies from evolutionary adaptiveness to everyday functioning that we considered above. As observed in the context of neural capacities for intrapersonal communication (e.g., the default-mode network), indirect measures may be informative such as with brain imaging. Cunningham (1992) reasonably resists pain perception as intrapersonal communication, but with physiological measurements and deliberate practices such as mindfulness meditation, this rejection of modalities beyond language could miss important nuances. It is likely that the active thoughts across and within individuals spans modalities, and indeed some report a total lack of inner speech (Nedergaard and Lupyan, 2024), making a more holistic view of this self-directed communication landscape necessary. A rough portrait of the potential complexity and multimodality of intrapersonal communication is depicted in Figure 1.

Below we discuss in more depth some of the existing overt measures that help to index and characterize intrapersonal processes, with an understanding that methodology can and should expand beyond these under the guidance of a unified study of intrapersonal communication.

#### 6.3.1 Natural language processing

While language may not tap into the rawest form of intrapersonal communication occurring internally in the form of thoughts, it can provide immense value in revealing information about our minds. Natural language processing (NLP) methodology continues to develop as computational tools increase in power and application (Jackson et al., 2022). NLP allows for the quantification of language through varying methods, including sentiment analysis, topic modeling, and more.

Among the simplest but most widely applied NLP techniques, Linguistic Inquiry and Word Count (LIWC) is a frequency-based word categorization library (Tausczik and Pennebaker, 2010). For the past few decades, LIWC has provided a method to study a wide range of linguistic functions, including negative and positive affective tone, temporal focus, and over a hundred other meaningful categories that have expanded with newer iterations. One study employing LIWC on recounts of trauma, told as though unfolding in real-time, found use of death-related words associated with more severe PTSD and depression, and poorer social adjustment after the traumatic event (Alvarez-Conrad et al., 2001). While these associations do not speak to causality, they showcase how word categories used in narratives, and other forms of verbal intrapersonal communication, can reveal mental-state information about the signaler.

LIWC can also analyze dynamics in the form of narrative arcs, such as how text flows across cognitive tension, staging, and plot progression. These measures reveal trends in different linguistic contexts, with unique signatures found for TED talks, newspaper articles, Supreme Court opinions, and narrative media (Boyd et al., 2020). These signatures of intrapersonal communication remain largely untapped, and will likely vary depending on whether the method of communicating involves unaltered streams-of-consciousness vs. more structured and edited journaling. On a much smaller scale, micro-features of real-time typing keystrokes may be meaningful as well. In a review of studies on typing dynamics and emotion detection, Maalej and Kallel (2020) reported that slower typing and higher error rates appeared to indicate negative emotions and stress. Linguistic work may also draw from mathematical concepts such as control theory, where desired behavior is achieved through a feedback loop that reduces discrepancies between a present state and desired state (Carver and Scheier, 1982).

Much of this work focuses on affective computing in the realm of human computer interaction (HCI), and with how much interpersonal communication now relies on digital formats, applications for computer-moderated intrapersonal communication may be just as fruitful.

#### 6.3.2 Spoken language

In cases where written or transcribed intrapersonal communication is available, NLP techniques can be immensely revealing. However, when intrapersonal communication is spoken, the additional signal provided by the voice itself can also provide valuable information, some of which may be unavailable or cumbersome to convey through typed language alone. Although often coded into data manually, partially internalized private speech is often whispered (Alarcón-Rubio et al., 2014), with whispers exhibiting both lower intensity and a lack of vocal fold vibration (Jovičić and Šarić, 2008). Some of this vocal information is likely the product of physiological influences that shapes vocal features by altering respiration or other production components (Scherer, 2003). High arousal emotions, such as anger, are likely to increase certain acoustic features, including intensity and fundamental frequency mean, variability, and range. In a study where nurses and nursing school students evaluated mock patient voice recordings, when the content of speech exhibited valence that was dissonant to the voice's tone (e.g., positive content with negative tone), the negative valence more strongly influenced perception and emotional responses (Yogo et al., 2000).

This finding suggests that analyses of content alone, such as transcriptions of speech, will not fully capture the emotional experience of spoken self-communicative instances. What specific features are necessary to infer emotion is unclear. Vocal and facial expressions can communicate a wealth of emotional information, including in contexts devoid of speech or other sources of semantic information (Bryant and Fox Tree, 2005; Laukka et al., 2013). Much of the work on vocal acoustics of emotions makes use of actors, which can call into question ecological validity of true emotional expressions as more variation is introduced through cultural influences (Bryant, 2021). However, in the case of intrapersonal communication, there could be benefits to understanding what features can be deliberately generated to volitionally signal favorable emotional states to achieve positive outcomes of overt spoken self-talk.

It might be the case that task performance and emotional regulation will benefit from different modalities or intrapersonal communication techniques, given the added emotional information accessible via overt private speech. A study looking at published essays from major figures and persuasive spoken public addresses found that the oral modality involved more personal references, more first and second person pronouns, and other features of word patterns and lengths (Einhorn, 1978). If persuasion techniques are different across modalities, it could be the case that self-persuasion operates differently orally vs. written as well.

#### 6.3.3 Think-aloud paradigm

One existing method for studying thoughts presented vocally is the think-aloud paradigm. Often used in the testing of computerized user interfaces, think-aloud involves speaking one's thought process aloud, typically as one performs a task such as navigating a website. Some scholars debate the validity of using such paradigms, often assuming the speech process disrupts the main task of interest, such as website navigation and perception (Cooke and Cuddihy, 2005).

Despite these concerns, think-aloud as a standalone paradigm might provide an informative window into intrapersonal communication. An association between negative, past-oriented, self-focused language, and rumination tendencies in spoken language (Raffaelli et al., 2021) appears to match similar findings in typed paradigms, which link “I-talk,” or self-focused language, to depression (Berry-Blunt et al., 2021). Some research also suggests overt vs. covert self-talk are equally beneficial for performance on tasks, although in a sample of patients with rheumatoid arthritis, written disclosure showed more potential influence than spoken disclosure (Lumley et al., 2011). Related research has used experience sampling in which a participant is prompted at regular intervals throughout a day to provide data (Van Raalte et al., 2019). This could help to evaluate mind-wandering tendencies (Stawarczyk et al., 2011). Different paradigms for eliciting self-talk may thus result in different styles of intrapersonal communication as well.

#### 6.3.4 Survey-based research

To understand the nature of intrapersonal communication, several questionnaire-based scales have been developed and tested. Some questionnaires examine the content of self-talk such as the Inner Speech Report, where one self-reports a list of as many topics of their self-talk as they can (Uttl et al., 2011). The Inner Speech Report parallels the thought-listing technique, which involves listing out all thoughts relating to some constraint, such as during a specific duration of time (e.g., “list all the thoughts you had while completing this past task”), and can include additional evaluations, such as reviewing one's own thoughts and tagging them for valence or other attributes (Cacioppo et al., 1997).

Other self-talk measures involve rating scales in response to targeted questions that provide context such as the Self-Talk Inventory, which presents imagined scenarios and asks how likely they would use different kinds of self-talk phrases (Uttl et al., 2011; Calvete et al., 2005). Uttl et al., for example, compared a collection of such self-report measures, finding internal consistency yet minimal evidence for validity across scales. Using such retroactive report style scales or paradigms are arguably indirect representations of more naturalistic and spontaneously occurring intrapersonal communication.

#### 6.3.5 Strengths and weakness of methodologies

A major barrier to establishing the phenomena of intrapersonal communication in research has been the wide-ranging collection of examples that potentially qualify. This problem is exacerbated by the limitations of methodologies as well. Currently, most related research appears focused on language, but the process of converting whatever endogenous communication is present into overt verbal communication could filter or otherwise alter the form. While spontaneously spoken thoughts used in think-aloud tasks might aim to reduce this conversion, it constrains thoughts into this particular modality and may force a linear trajectory onto thoughts that is not organic. Thought-listing can eliminate the linear trajectory by allowing any and all thoughts be shared (even if they may have co-occurred), the retrospective aspect may lose accuracy of reports, and the act of listing these thoughts in itself represents a stream of information, where new ideas could falsely be included. Even if these limitations are set aside, the use of natural language processing techniques to infer function within form, such as assigning positive or negative sentiment, assume generalizability. To study something as complex as our inner communication landscape, it is necessary to better integrate a wider range of methods, one of the benefits that could emerge from a dedicated study of intrapersonal communication.

In addition to the methods described above, new empirical insights could be gained through dynamic measurements, such as neuroimaging, physiological measurements, eye-tracking, body-tracking, and more. These dynamic techniques could be used in conjunction with most of the methods reviewed above. Though it is outside the scope of the present review, interested readers can consult theoretical and methodological reviews of such continuous measures in Spivey and Dale (2004), Spivey (2008), and Favela (2020).

## 7 Concluding remarks

By taking a multi-dimensional approach to intrapersonal communication, more can be understood about the dynamics of self-awareness in human experience. Future work would benefit from fleshing out the space intrapersonal communication encompasses, considering a wider spectrum of conscious and unconscious variations. This work would build on themes such as context (Brinthaupt et al., 2015), content, and modality, while identifying possible dimensions that determine how our self-influence plays out, such as awareness, attention, or perhaps vividness of the endogenous experience. By incorporating work on mindfulness with an intrapersonal communication framework, a unified study could then investigate the ways active and inactive sender and receiver roles within the self shape our experiences with differing levels of success or salience. Methods, theories, and findings from different relevant fields could be leveraged by each other to delve deep into the potential that various intrapersonal communication behaviors can have on things such as education and mental wellbeing.

As raised by Cunningham (1992), intrapersonal communication as an area of study risks an extreme hypothetical perspective that requires a private language for inner experiences, or at another extreme include the entirety of information within an individual, making the distinction between intrapersonal communication and cognition unclear. The greatest challenge for this area of study remains the defining of meaningful boundaries. The proposed active/inactive sender-receiver framework provides one approach to boundaries by characterizing the active involvement in the internal messages as the defining dimension. The process of activating any communication role within the self may be enough to qualify a signal as intrapersonal communication. However, the case of the self as both inactive sender and inactive receiver leaves ambiguities unaddressed: How should basic cognition be factored into definitions of intrapersonal communication, if at all? Other frameworks may also privilege other criteria over active engagement in these sender and receiver roles. If an internal signal is perceivable but volitional control is limited or nearly inaccessible, such as with auditory hallucinations or dreams without lucidity, then this boundary could remain debatable. However, many of the limitations for the proposed framework are similar to those shared across all communication frameworks.

In intrapersonal communication, we must first consider the scope that defines the “personal” or meaningful entity that this communication occurs internal to. From there, we can focus on intrapersonal communication in the domain of human individuals, though future work should certainly extend beyond humans, considering non-human animals and artificial intelligences. To better understand intrapersonal communication and the forms and functions that occur, frameworks such as the proposed active/inactive sender-receiver model can guide categorization and inform possible boundaries or limitations. Other possible frameworks could consider characterizing a hierarchy of intrapersonal communications along dimensions such as unconscious-conscious levels, intended-unintended, vividness, and so on. From there, clarification of these mechanisms, their functional potential, and the boundaries around what is and is not intrapersonal communication, may become possible.
